# Detection of Metalloproteases and Cysteine Proteases RNA Transcripts of *Leishmania (Leishmania) infantum* in Ear Edge Skin of Naturally Infected Dogs

**DOI:** 10.1155/2020/2615787

**Published:** 2020-06-24

**Authors:** Laura Barral Veloso, Flávia de Oliveira Cardoso, Karen dos Santos Charret, Matheus Pereira de Sá Silva, Luzia Monteiro de Castro Côrtes, Kátia da Silva Calabrese, Franklin Souza da Silva, Joel Fontes de Sousa, Marília Fonseca Rocha, Carlos Roberto Alves

**Affiliations:** ^1^Fundação Oswaldo Cruz, Instituto Oswaldo Cruz, Laboratório de Biologia Molecular e Doenças Endêmicas, Avenida Brasil, 4365, CEP 21040-900, Manguinhos, Rio de Janeiro, RJ, Brazil; ^2^Fundação Oswaldo Cruz, Instituto Oswaldo Cruz, Laboratório de Imunomodulação e Protozoologia, Avenida Brasil, 4365, CEP 21040-900, Manguinhos, Rio de Janeiro, RJ, Brazil; ^3^Fundação Oswaldo Cruz, Centro de Desenvolvimento Tecnológico em Saúde, Avenida Brasil, 4365, CEP 21040-900, Manguinhos, Rio de Janeiro, RJ, Brazil; ^4^Centro de Controle de Zoonoses-Prefeitura Municipal de Montes Claros-Avenida Antonio Lafetá Rebelo, 1371, CEP 39402-089, Santa Lucia II, Montes Claros-MG, Brazil

## Abstract

*Leishmania* spp. proteases have been proposed as virulence factors contributing to adaptive success these parasites to the mammalian hosts. Since these enzymes are poorly studied in naturally infected dogs, this work aims to show the differences in metalloprotease and cysteine proteases gene expression in ear edge skin of dogs naturally infected by *Leishmania (Leishmania) infantum*. A cohort of dogs (*n* = 20) naturally infected by *L. (L.) infantum* was clinically classified as asymptomatic, oligosymptomatic, and polysymptomatic and the parasite load range estimated. The analysis of proteases expression by RT-PCR in the ear edge skin was also assessed, suggesting more transcripts of proteases in cDNA samples from polysymptomatic dogs than oligosymptomatic and asymptomatic ones. Metalloprotease RT-PCR assays yielded products (202 bp) in all assessed cDNA dog samples. In contrast, cysteine proteases transcripts (227 bp) had shown to be better detected in cDNA samples of polysymptomatic dogs, compared with cDNA samples from asymptomatic and oligosymptomatic dogs. Predictive *in silico* assays suggested that secondary structures of metalloproteasee mRNAs can be more stable than cysteine proteases at the skin temperature of dogs. Evidence is presented that during natural infection of dogs by *L. (L.) infantum*, this parasite produces transcripts of metalloprotease and cysteine protease RNA in the skin from asymptomatic, oligosymptomatic, and polysymptomatic dogs.

## 1. Introduction

Leishmaniasis is a vector-borne disease in the Americas, Africa, Mediterranean basin, Western and Central Asia, India, and Australia (http://www.who.int/en/—accessed in 02-20-2020); it is caused by different species of digenetic protozoan parasites belonging to the genus *Leishmania*. In their life cycle, these parasites live as extracellular promastigotes inside the insect vector gut, and as intracellular amastigotes in mammalian host cells, especially phagocyte mononuclear system cells [1, 2]. There are two main clinical forms: cutaneous leishmaniasis (CL) and visceral Leishmaniasis (VL), in humans and animals such as dogs, rodents, and marsupials [3].

In endemic areas of the world, thousands of dogs are infected by *Leishmania* (*Leishmania*) *infantum*, affecting both the viscera and skin—canine leishmaniasis (CanL) [4]. In Brazil, *L.* (*L.*) *infantum* is described as the main agent of canine visceral leishmaniasis (CanVL) and is associated with cases of human VL [5]. Canine visceral leishmaniasis clinical profile is characterized by different features, ranging from asymptomatic to symptomatic dogs, which may follow at least one of these signs, alopecia, apathy, cutaneous lesions, diarrhea, fever, lymphadenopathy, onychogryphosis, pale mucous membranes, polyuria, polydipsia, splenomegaly, and vomiting [6]. The clinical manifestations of CanVL may be resulting from interactions of *L. (L.) infantum* of the reservoir host immune system status and virulence factors, as proteases. These enzymes are well understood in mice model and in human infections; however, they are poorly studied in canine infections [7].

According to several *Leishmania* spp. proteases studies, these enzymes are essential for different activities such as tissue invasion, modulation on the host immune response [7] in the pathogenesis of infection [8]. Under their regulatory roles that can be related to four main different protease classes, which are classified as cysteine proteases, serine proteases, aspartic proteases, and metalloproteases. The first three enzymes are classified according to amino acid residue as serine, cysteine, or aspartic acid in the active site, and the metalloproteases use a metal ion for catalytic activity [9].

The proteases' specific role in *Leishmania* spp. are known to be essential for its life cycle [10–13]. The higher expression of these genes in amastigotes is an important evidence of these enzymes on the evolutionary cycle stage in vertebrate hosts because of the new metabolic demand and escape mechanisms in hosts [7, 14]. Although, protease genes are present on all chromosomes in at least four different *Leishmania spp.*: *L. (Viannia) braziliensis*, *L. (L.) infantum*, *L. (L.) major*, and *L. (L.) mexicana* occurring in different amounts [15]. This is consistent with the already reported importance of proteases for these parasites, as it reveals that encoded genes for these enzymes are abundantly dispersed among the *Leishmania* spp. genomes. It is an indication that distinct patterns of protease evolution have affected the different *Leishmania* spp. and for consequence having biological effects in their hosts.

Thus, this work aims to add new information on the metalloproteases and cysteine proteases gene expression of *L. (L.) infantum* in ear edge skin of infected dogs with this parasite. These enzymes have not yet been adequately described in naturally infected dogs and for the first time proposed evidence of both enzyme in different clinical cases of infected dogs.

## 2. Materials and Methods

### 2.1. Chemicals and Culture Media

Penicillin, streptomycin, Schneider's Drosophila medium, cell culture flasks, and the fluorescent stain Nancy-520 were purchased from Sigma-Aldrich Chemical Co. (St. Louis, MO). Fetal calf serum (FCS), primers for proteases, kDNA3 and housekeeping genes, TRIzol reagent, RNase H enzyme, DEPC-treated water, deoxyribonucleotide phosphate solution (dNTPs), Platinum Taq DNA Polymerase (DNA Polymerase), and Taq Platinum PCR buffer were purchased from Invitrogen, Life Technologies (USA). iScript cDNA Synthesis kit was purchased from Bio-Rad Laboratories, (Hercules, CA). GoTaq® qPCR Master Mix, Wizard SV Gel Kit, and PCR Clean-UpSystem were purchased from Promega Corporation (USA). High Pure PCR Template Preparation Kit was purchased from Roche Molecular Systems, Inc. Chloroform and ethanol were purchased from Merck (Brazil). All reagents were of analytical grade or higher.

### 2.2. Ethics Statement

Dog tissues were provided by Municipal Zoonotic Diseases Control Department (ZDCD) of Montes Claros/MG, from January 2016 to December 2017 as a result of the Disease Control Program, according to the norms and guidelines of the Brazilian Ministry of Health—Surveillance, Prevention and Control Manual (2016). All collections followed the current procedures for confirmed disease cases and with permission of the animal's guardian, according to Resolution No. 1000 of May 2012—Federal Council of Veterinary Medicine.

### 2.3. Parasites and Culture Conditions

*Leishmania* (*Leishmania*) *infantum* (strain MHOM/TN/1993/LV10) strain was obtained from the *Leishmania* collection of the Instituto Oswaldo Cruz (CLIOC), maintained as promastigotes in semisolid NNN medium at 26°C. Culture expansion was performed in Schneider's insect medium at pH 7.2 (supplemented with 10% FCS, 200 IU penicillin, 200 *μ*g/mL streptomycin) and maintained at 26°C.

### 2.4. Dog Sample Tissues

For this study, tissue samples were collected from 20 mongrel dogs, with unknown age. The animals were identified as naturally infected by *Leishmania* during the seroepidemiological survey for CanL, carried out by the ZDCD, and destinated to compulsory euthanasia. All animals had presented positive results for CanVL based on two serological tests (rapid DPP CanVL screening test and EIE CanVL confirmatory test) and PCR (unpublished results). Then, these animals were classified in three different groups by their clinical signs: asymptomatic (As), dogs without any apparent clinical sign; oligosymptomatic (Os), dogs with up to two clinical signs; polysymptomatic (Ps), dogs that presented three or more typical clinical signs for CanL. After euthanasia, postmortem biopsy fragments (3 mm) from the ear edge tissues were collected, using a dermatological punch, washed and stored in TRIzol reagent at -80°C.

### 2.5. DNA and RNA Extraction and Purification

For DNA extraction, tissue fragments and promastigote cultures were processed with High Pure PCR Template Preparation Kit following the manufacturer's instructions. Total RNA from tissues and promastigotes cultures was extracted using TRIzol reagent following the manufacturer's instructions. The concentration and quality of DNA/RNA samples were determined using a Pico200 microliter spectrophotometer (PicodropLtd., Saffon Walden, UK) and stored at -80°C. cDNA synthesis was performed with total RNA (1 *μ*g) using iScript cDNA Synthesis kit according to the manufacturer's recommendations.

### 2.6. Sequences Selection and Primers Design

Primers that amplify regions with 100% identity and similarity between the sequences of metalloproteases (202 bp) and cysteine proteases (227 bp) of *L.* (*L.*) *infantum* target genes, *α*-tubulin (160 bp) and *β*-actin dog genes (87 bp) were generated after *in silico* analysis of these regions using Trytrip databank (http://tritrypdb.org/tritrypdb/) to obtain the sequences. Sequence alignments were performed on ClustalW2 webserver (https://www.ebi.ac.uk/Tools/msa/clustalw2/). The initiators for protease genes were designed by Primer3Input (http://bioinfo.ut.ee/primer3-0.4.0/), with all parameters set to default except the product size ranges. Primer targeting *Leishmania* kinetoplast minicircle 3 (kDNA3; 120 bp) was synthesized as previously reported [16]. The primers were synthesized the scale of 50 nM and purified by desalting. All primer sequences are in Table 1.

### 2.7. Quantitative Polymerase Chain Reaction (qPCR) Assay Conditions

Reaction mixtures for the *Leishmania* kDNA quantification contained 10 *μ*L of GoTaq® qPCR Master Mix, kDNA3 (200 nM) or *α*-tubulin primers (200 nM) and 5.0 *μ*L of DNA template (25 ng); the final reaction volume was adjusted to 20 *μ*L with H_2_O. PCR conditions: on step (95°C for 2 min), followed by 40 cycles (95°C, 15 s; 60°C, 1 min). Standard curves were generated from 10-fold serial dilutions of *Leishmania* DNA. A melt curve analysis was performed for all reactions. The quality parameters of the standard curves were analyzed with the QuantStudio™ Design & Analyss Software v1.4.3 (Applied Biosystems, Foster City, California, USA). *α*-tubulin reference gene was used as positive control to monitor DNA integrity, presence of potential inhibitors of PCR or variation in DNA yield. Quantification of parasites in fragments from the ear edge was conducted using QuantStudio 3 Real-Time PCR System.

### 2.8. PCR Assays for Metalloprotease and Cysteine proteases

Differences in the mRNA expression of metalloproteases and cysteine proteases were assayed for PCR in fragments from the ear edge skin as follows: (i) master mixes (1 mM Tris-HCl, pH 8.3, 50 mM KCl, 4 mM MgCl2, 2.5 mM of each dNTP, 10 *μ*M of each primer and 1 U Taq DNA polymerase with 100 ng of cDNA and adjusted to a final volume of 25 *μ*L); (ii) amplification cycle conditions (94°C, 5 min) followed by 30 cycles (95°C, 45 s; 60°C, 45 s; 72°C, 90 s) and a final elongation step (72°C, 5 min) using a Veriti ThermalCycler (Applied Biosystems, Foster City, CA, USA). The amplified products (10 *μ*L) were evaluated by 2.0% (*w*/*v*) agarose gel electrophoresis and stained with Nancy-520.

### 2.9. Sequencing of Protease Gene PCR Product

PCR products were purified with Wizard® DNA Clean-Up System, following the manufacturer's protocol and quantified in a Nanodrop 2000c. After that, PCR product (200 ng) and forward primer (200 ng) for the studied genes were used for sequencing using a Sequence Scanner (Applied Biosystems). The results were analyzed in the BioEdit program (Ibis Biosciences, Carlsbad, CA, USA).

### 2.10. Calculation of the mRNAs Folding Analysis

The protease gene sequences for *Leishmania (L.) infantum* (Gene ID: LinJ 10 0501, LinJ 10 0510, LinJ 08 0960) and *Leishmania (V.) brasiliensis* (Gene ID: LbrM 08 0810, LbrM 10 0610, LbrM 10 0590, LbrM 08 0820, LbrM 08 0830) were retrieved from TriTrypDB server (https://tritrypdb.org/tritrypdb/) obtaining the respective mRNA sequences. After that, the predicted two-dimensional (2D) models of mRNAs were obtained using mfold web server (http://unafold.rna.albany.edu/?q=mfold/RNA-Folding-Form). The mRNAs folding was assayed at different temperatures (26°C to 40°C). These structures were analyzed using single-stranded value (ss-value), which determines the probability of linearity of the structure. Thus, the higher value of ss-value, the greater the probability to have single strands in the 2D RNA structure.

## 3. Results

### 3.1. Parasite Load

The characterization of the selected cohort (*n* = 20) was performed based on parasite load; it was estimated from kDNA-qPCR analysis of the asymptomatic, oligosymptomatic, and polysymptomatic dogs. The performance of qPCR for *Leishmania* kDNA quantification was related to the presence of the parasite in the ear edge skin and defined as parasite load.

**Figure 1:**
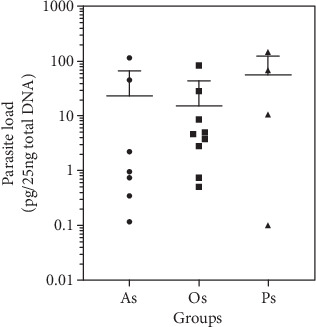
Quantification of *Leishmania* kDNA by real-time PCR assay (qPCR). Parasite load in ear edges skin of dogs naturally infected with *Leishmania (L.) infantum* was assessed in asymptomatic (As), oligosymptomatic (Os), polysymptomatic (Ps) dogs. Results are expressed (pg kDNA/25 ng total DNA) as the mean ± SD of two independent experiments realized in triplicate.

The results had shown an extensive range of parasite load in the ear edge skin, from 0.10 ± 0.01 to 146.9 ± 2.65 pg (kDNA)/25 ng of total DNA on those three dog groups (Figure 1). Asymptomatic dogs showed a parasite load between 0.1 ± 0.01 and 115.0 ± 5.4 pg (kDNA)/25 ng of total DNA, oligosymptomatic dogs from 0.7 ± 0.05 to 83.5 ± 5.0 pg (kDNA)/25 ng of total DNA, and polysymptomatic dogs from 0.1 ± 0.01 to 146.9 ± 2.7 pg (kDNA)/25 ng of total DNA. Even though, there were not detected significant differences between the clinical groups of dogs, these data suggest higher parasite load mean in the polysymptomatic group.

### 3.2. Parasite Protease Genes Expression

The course of this work assessed the metalloproteases and cysteine proteases genes expression in tissues from asymptomatic, oligosymptomatic, and polysymptomatic dogs. The predicted amplified sequences were confirmed by conventional PCR assays. As expected, the designed primers were specific for conserved regions of each protease gene amplified, metallo and cysteine proteases of *L.* (*L.*) *infantum* parasites. The sequencing of each amplicon resulted in compatible sequences for expected target sequences, which was confirmed by performing alignments with BLAST software against public databases (Supplementary file 1).

**Figure 2:**
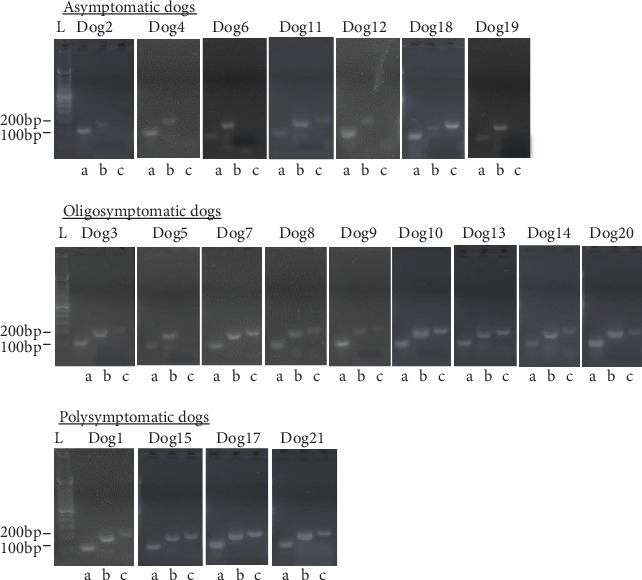
Expression of metalloprotease and cysteine protease genes from *Leishmania* (*L.*) *infantum* in naturally infected dogs. Total RNA from dog's ear edge skin was extracted and reverse transcription polymerase chain reactions (RT-PCR) were performed using specific primers for the *β*-actin (a), metalloprotease (b), and cysteine protease (c) genes. RT-PCR products were resolved on a 2% agarose gels stained with Nancy-520. A 100 bp DNA ladder (L) was used as a molecular weight marked and revealed single 87 bp, 202 bp, and 227 bp fragments, respectively, in the tested cDNA samples. Asymptomatic (Dog 2, Dog 4, Dog6, Dog 11, Dog 12, Dog 16, and Dog 19), oligosymptomatic (Dog 3, Dog 5, Dog 7, Dog 8, Dog 9, Dog 10, Dog 13, Dog 14, and Dog 20) and polysymptomatic (Dog 1, Dog 15, Dog 17 and Dog 21).

In the following phase of the study, ear edge skin from asymptomatic, oligosymptomatic, and polysymptomatic dogs positive for CanVL was assessed for proteases transcripts detection. The PCR product bands were better detected in polysymptomatic dog cDNA samples than oligosymptomatic and asymptomatic dogs (Figure 2). In general, metalloprotease RT-PCR assays yielded products (202 pb) in all assessed cDNA samples, suggesting more transcripts of these genes in polysymptomatic dog samples. Conversely, transcripts of cysteine-proteases genes (227 pb) had shown bands in cDNA samples from polysymptomatic and oligosymptomatic dogs, which were slightly detected in cDNA samples from the asymptomatic dogs (Figure 2). Additionally, the *β*-actin dog gene (87 pb) was included in this study as a constitutive gene for positive control in all assayed samples.

### 3.3. Predicting Secondary Structure of Protease mRNAs of the Leishmania spp. Induced by Temperature Change

**Figure 3:**
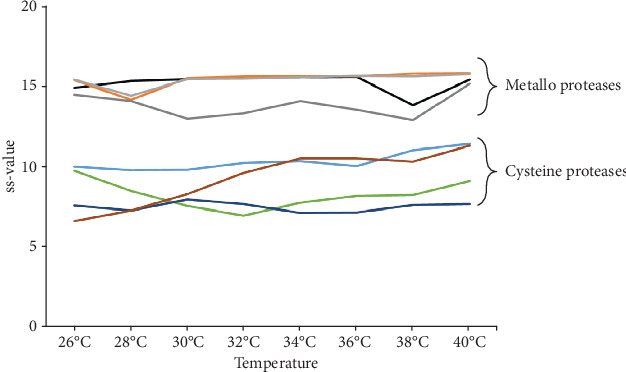
*Leishmania* spp. proteases mRNA folding stability. The two-dimensional mRNA structures were calculated by using “The mfold Web Server” assessing at different temperature: 26°C, 28°C, 30°C, 32°C, 34°C, 36°C, 38°C, and 40°C. RNA folding stability was expressed as measured propensity of a base to be single-stranded (ss-value). The calculations were performed for mRNA sequences of metalloproteases and cysteine proteases.

The stability prediction of two-dimensional mRNA structures of metalloproteases and cysteine proteases, between temperatures ranging from 26°C to 40°C, was obtained by calculating the ss-values and *Δ*G values, (Figure 3, Supplementary file 2 and 3). These assays were performed with the protease mRNA sequences compatible with the expected target sequence for the designed primer to RT-PCR assays (Table 1).

The highest values with the smallest ss-value variations were related to the smallest variation in two-dimensional mRNA structures of metalloproteases (LinJ 10 0501: ss − value = 15.2 ± 0.6; LinJ 10 0510 and LbrM 10 0610: ss − value = 15.4 ± 0.5; LbrM 10 0590: ss-value 13.8 ± 0.8). On the other hand, two-dimensional mRNA structures of cysteine proteases had shown the lowest values with the highest ss-value variations (LbrM 08 0810: ss − value = 8.3 ± 0.8; LbrM 08 0820: ss − value = 7.5 ± 0.3; LbrM 08 0830: ss − value = 9.3 ± 1.5; LinJ 08 0960 ss − value = 10.3 ± 0.6).

Additionally, the data of mRNA folding suggested that secondary structures predicted for cysteine protease mRNAs may show the lowest *Δ*G values, between the temperature of 26°C and 40°C (LbrM 08 0810: -626.99 and -456.28; LbrM 08 0820: -618.96 and -450.41; LbrM 08 0830: -493.69 and -518.16; LinJ 08 0960: -493.69 and -538.16) than *Δ*G values for metalloproteases mRNA (LinJ 10 0501: -718.04 and -752.74; LinJ 10 0510: -715.57 and -749.53; LbrM 10 0610:-691.68 and -725.89; LbrM 10 0590: -701.68 and -734.23) (Supplementary file 2 and 3).

## 4. Discussion

Proteases have been described as virulence factors of *Leishmania* spp., related to the adaptive success of these parasites in the vertebrate hosts. The main studies regarding the role of proteases in the vertebrate hosts are extensively related to infections from mice-model and humans [7], and there are few studies with those enzymes in dogs with CanVL, which are the focus of this study.

The selected cohort of dogs was previously diagnosed as CanVL by *L*. (*L*.) *infantum*. This previous characterization was the criteria for selecting the dogs to assess protease genes. The data presented here, concerning the presence of the parasite load in the dogs' skin, are in comparison with the data previously described in the literature, which describes the polysymptomatic dogs with the highest parasitic loads followed by the oligosymptomatic and after the asymptomatic ones [17, 18].

In the course of this work, mRNA of *L*. *(L.) infantum* proteases were accessed in the tissues of canine cohort. Evidence is gathered that metalloproteases and cysteine proteases of the parasite have differential expression in ear edge skin of naturally infected dogs (asymptomatic, oligosymptomatic, and polysymptomatic) from an endemic area. The data indicate that factors such as cDNA yield, purity, and mRNA extraction method did not affect PCR sensitivity on assays for all assessed dog samples, providing more reliable interpretation of RT-PCR results and contributing to the quality of these experiments [19–21].

It is known that virulence factors of *Leishmania* spp. determine their pathogenicity. Studies on virulence factors of these parasites favor ample space to identify factors or cofactors that contribute to the pathogenesis outcome. Including proteases that have been reported to be active in various stages of *Leishmania* spp. infection, such as tissue invasion, macrophage survival, and host immune response modulation [7]. Enriching the knowledge about proteases as virulence factors of the parasites, this work showed that metalloproteases are expressed in the ear edge skin of infected dogs from an endemic area. Interestingly, our data suggest that cysteine proteases, unlike metalloproteases, are downregulated in asymptomatic and oligosymptomatic dogs infected with *L. (L.) infantum.*

The findings of this work regarding the unrestricted presence of metalloprotease transcripts in the ear edge skin of dogs infected with *L.* (*L.*) *infantum* agree with the knowledge of the participation of these enzymes in the course of infection of vertebrate hosts, contributing for the pathogenesis. Although, the information on metalloproteases as parasite virulence factors in dog is poorly explored, the direct involvement of these proteases in the parasite-host interaction has been proposed for the mammalian hosts in general. Metalloproteases, named Gp63, have the largest studies described in the host-parasite interaction, helping to keep the parasite in the hostile host environment [7].

Members of metalloprotease classes have been involved in tissue invasion, immunoglobulin G hydrolysis, cleavage and inactivation of complement factor C3b, adhesion, and internalization in macrophages [7]. Decreased gp63 expression in mice induces Th1 profile as cleavage of transcription factor NF-*κ*B interfering with mouse IL-12 and iNOS expression [22, 23]. Other studies indicate that metalloproteases (63 kDa), or Gp63, cleaves CD4 on human T cells [24] and inhibit the proliferation of natural killer cells [25]. In murine bone marrow macrophages, Gp63 interferes on signaling cascade and affects transcription factors by c-Jun cleavage of the central component of AP-1 [26].

The low detection of cysteine protease genes can be explained, because these proteases influence parasite-host interaction interfering in host immune response. These proteases interfere in antigen presentation by cells due to cleavage of MHC class II proteins in mice [27] and are able to induce a Th2 profile in BALB/c mice increasing lesions with IL-4 and IL-5 production [28–31]. The inhibition of nitric oxide production by the signal transducer and transcription activator 1 cleavage are also related with cysteine protease, as well as the induction Th1 profile in C3HeB/FeJ and C57BL/6 mice due to response-associated cytokines [28, 32–35]. Cysteine protease epitopes modulate infection in BALB/c and CBA mice by inducing Th1 and Th2 response-related cytokines with CD8^+^ T lymphocyte stimulation [36, 37], having roles in the intracellular survival of the parasite and in its interaction with its mammalian host [38]. Additionally, the contribution to the intracellular survival of the parasite within the host macrophages by activating latent TGF-beta1 was related to these enzymes [39].

Some cysteine protease actions can be affected by the differential regulation of these enzymes genes, since they are originated in the same transcriptional unit suggesting large differences in the expression pattern in *L.* (*L.*) *infantum* [40]. As previously suggested, the increase of temperature can be an inductor control factor of *cpb* genes expression influencing regulatory elements that accelerate RNA degradation [41].

Thus, the participation of metalloproteases and cysteine proteases in canine infection can be related to the respective expression profile in dog tissue. This hypothesis is reinforced here by performing in silico analysis, which had shown differences to two-dimensional structure propensity of protease mRNAs assessed; measured by the number of times is single-stranded in a group of predicted folding, according to temperature variation. Therefore, the data showed that the mRNA two-dimensional structures of metalloproteases can be more stable than the mRNA two-dimensional structures of cysteine proteases between the temperature of 26°C and 40°C. Interestingly, the sequence of the expected mRNA of LbrM 08 0830 gene was detected more instability of two-dimensional structure as temperature increased, compared with other cysteine protease genes. Additional assays are necessary to prove the two-dimensional mRNA structure in the regulation of these genes. It is known that parasite mRNA structure changes can be a mechanism for expression and activity control of its proteases, as previously proposed by *in vitro L.(V.) braziliensis* differentiation due to temperature changes [41].

Thus, the parasites present in dog skin might have a differential regulation of metalloprotease and cysteine protease gene expression. Possibly, the mRNA expression of both proteases can be controlled by skin temperature-once the median axillary temperature in dogs is 38.4°C [42]. Furthermore, the results of the present work suggest possibilities of action of these enzymes in the dogs' pathogenesis balance status, influencing the emergence of signs and symptoms of the CanL meaning a parasite strategy to keep vertebrate host infection. However, further studies are needed to substantiate this statement.

## 5. Conclusion

The results presented here are evidence that in natural dog infection, the parasite *L*. (*L*.) *infantum* has an active expression of proteases in the skin of asymptomatic, oligosymptomatic, and polysymptomatic dogs. The metalloproteases of this parasite are expressed at the ear edge, regardless of the animal's clinical profile, whereas cysteine protease transcripts are more related to the disease clinical form, being more evident in polysymptomatic dogs.

## Figures and Tables

**Table 1:** Pairs of primers designed for the analysis of the expression of target genes for *Leishmania spp.* and dog using PCR assays.

	GeneDB ID	Gene-target	Primers foward/reverse	Product size (pb)
*Leishmania* spp. genes	LinJ.10.0501	Metallo protease: gp63, leishmanolysin	5′ GGGTAGGGGCGTTCTGC 3′ 5′ CCCGGCCTATTTTACACCAACC 3′	202
	LinJ.10.0510			
	LbrM.10.0610			
	LbrM.10.0590			
	LbrM.08.0810	Cysteine protease: cathepsin L-like protease	5′ GGGTAGGGGCGTTCTGC 3′ 5′ CCCGGCCTATTTTACACCAACC 3′	227
	LbrM.08.0820			
	LbrM.08.0830			
	LinJ.08.0960			
	M94088	Kinetoplast minicircle 3 (kDNA3)	5′ CTGATCCACTGTTTTCTCCCCA 3′ 5′ AAAGTGCCCGTGAGTACAGG 3′	120

*Canis lupus familiaris*	LOC106557476	*α*-Tubulin	5′ GGGTAGGGGCGTTCTGC 3′ 5′ CCCGGCCTATTTTACACCAACC 3′	160
	XM_003124280.5	*β*-Actin	5′ CTGATCCACTGTTTTCTCCCCA 3′ 5′ AAAGTGCCCGTGAGTACAGG 3′	87

## Data Availability

The data obtained with NCBI BLAST tool and of the mRNA bidimensional structure of Leishmania spp. protease used to support the findings of this study are included within the supplementary information file.

## Supplementary Materials

Supplementary Materials Supplementary material 1: analysis of PCR products performed with protease primers using the NCBI BLAST tool. Sequencing results of each amplicon showing compatible sequences for expected target sequences, which was confirmed by performing alignments with BLAST software against public databases. Supplementary material 2: plotting of bidimensional structure of Leishmania spp. metalloprotease mRNAs according to temperature. The stability prediction of two-dimensional mRNA structures of metalloproteases, between temperatures ranging from 26°C to 40°C, was obtained by calculating the ss-values and *Δ*G values. Supplementary material 3: plotting of bidimensional structure of Leishmania spp. cysteine protease mRNAs according to temperature. The stability prediction of two-dimensional mRNA structures of metalloproteases, between temperatures ranging from 26°C to 40°C, was obtained by calculating the ss-values and *Δ*G values.
