# Supplementary material for: Detection of Metalloproteases and Cysteine Proteases RNA Transcripts of Leishmania (Leishmania) infantum in Ear Edge Skin of Naturally Infected Dogs
Source: Biomed Res Int. 2020 Jun 24;2020:2615787. doi: 10.1155/2020/2615787 (PMC7333044; doi:10.1155/2020/2615787)
Supplement: Supplementary Materials — Supplementary material 1: analysis of PCR products performed with protease primers using the NCBI BLAST tool. Sequencing results of each amplicon showing compatible sequences for expected target sequences, which was confirmed by performing alignments with BLAST software against public databases. Supplementary material 2: plotting of bidimensional structure of Leishmania spp. metalloprotease mRNAs according to temperature. The stability prediction of two-dimensional mRNA structures of metalloproteases, between temperatures ranging from 26°C to 40°C, was obtained by calculating the ss-values and ΔG values. Supplementary material 3: plotting of bidimensional structure of Leishmania spp. cysteine protease mRNAs according to temperature. The stability prediction of two-dimensional mRNA structures of metalloproteases, between temperatures ranging from 26°C to 40°C, was obtained by calculating the ss-values and ΔG values. [file 2615787.f1.docx]

**Supp 1:** Analysis of PCR products performed with protease primers using the NCBI BLAST tool

| **Sample** | **Description** | **Max Score*** | **Total score**** | **Query cover (%)****** | **E value****** | **Ident (%)******* |
| --- | --- | --- | --- | --- | --- | --- |
| *Leishmania braziliensis* DNA | *Leishmania braziliensis* MHOM/BR/75/M2904 cathepsin L-like protease (CPB), partial mRNA | 287 | 287 | 57 | 2e-73 | 96.05 |
| *Leishmania braziliensis* DNA | *Leishmania braziliensis* partial gp63 gene for glycoprotein, isolate MHOM/BR/75/M2903, clone 10 | 226 | 226 | 46 | 5e-55 | 92.12 |
| *Leishmania infantum* DNA | [*Leishmania infantum* JPCM5 cathepsin L-like protease partial mRNA](https://blast.ncbi.nlm.nih.gov/Blast.cgi#alnHdr_339896952) | 346 | 346 | 63 | 3e-91 | 99.48 |
| *Leishmania infantum* DNA | [*Leishmania infantum* surface glycoprotein gp63 (gp63) gene, complete cds](https://blast.ncbi.nlm.nih.gov/Blast.cgi#alnHdr_1213329) | 183 | 183 | 48 | 3e-42 | 86.63 |

(*****) **Max Score:** refers to the longest aligned fragment; (******) **Total Score:** refers to the total aligned sequence; (*******) **Query cover:** refers to the percentage of the query sequence that overlaps the reference sequence; (********) **E-value:** refers to the number of expected hits of similar quality that could be found just by chance; (*********) **Identity:** refers to the percent that describes how similar the query sequence is to the target sequence.


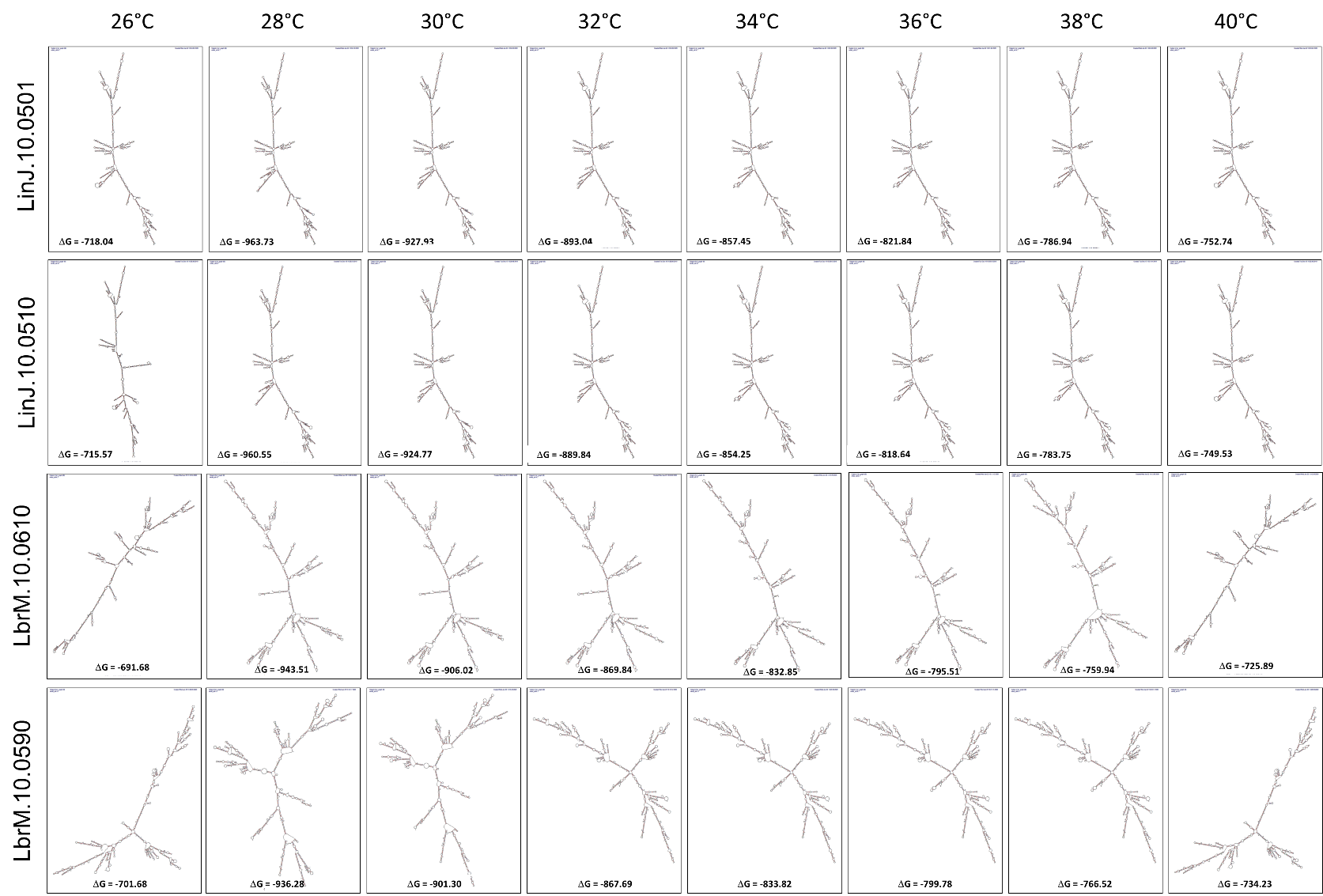


**Supp 2:** **Plotting of bidimensional structure of *Leishmania* spp. metallo protease mRNAs according to temperature**. The sequences of each of the genes were analyzed using the Una fold.rna platform (<http://unafold.rna.albany.edu/?q=mfold/RNA-Folding-Form>), where 2D structures were obtained. The structures are formed according to the rule of Gibbs free energy (ΔG).


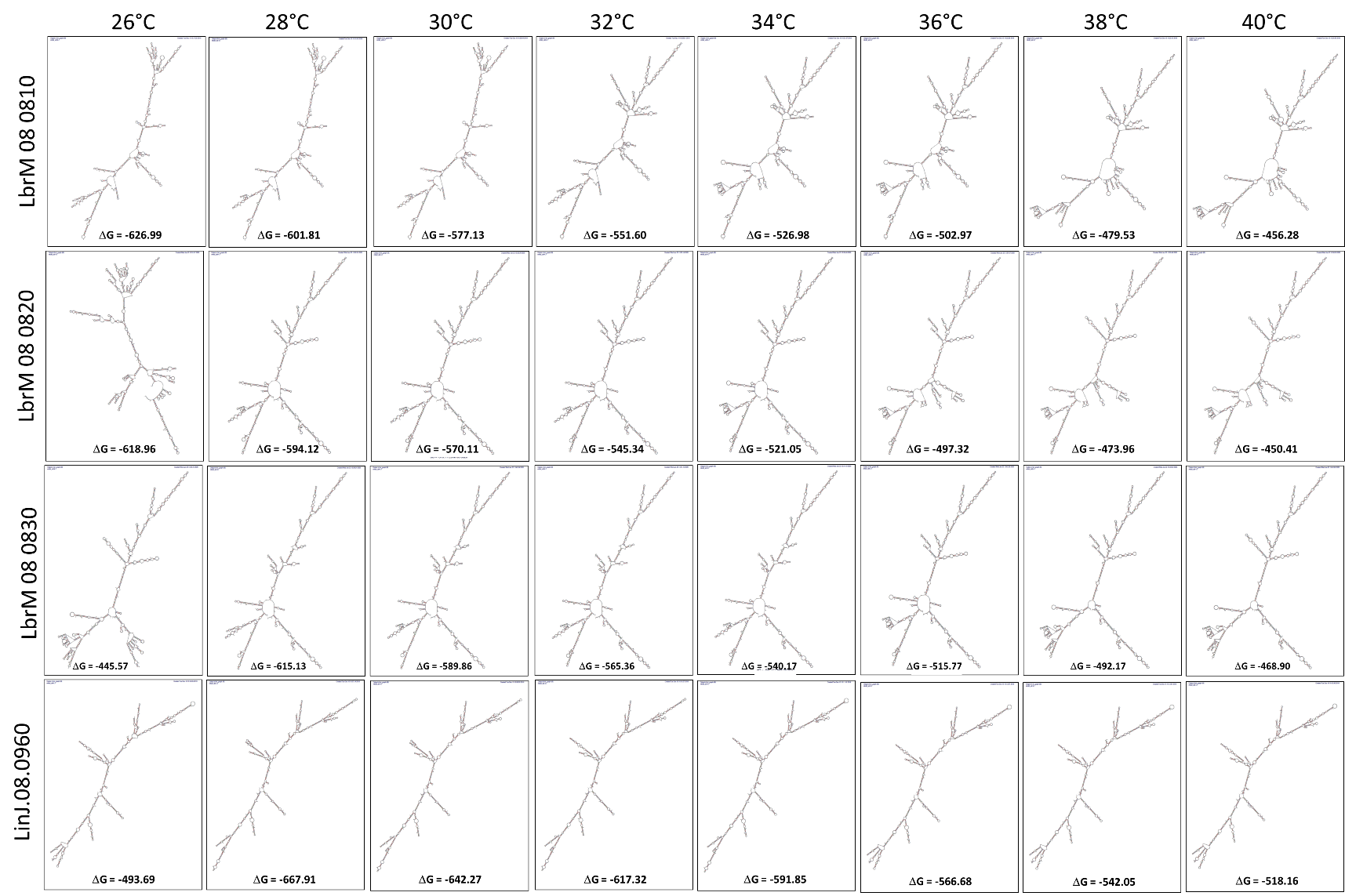


**Supp 3:** **Plotting of bidimensional structure of *Leishmania* spp. cysteine protease mRNAs according to temperature.** The sequences of each of the genes were analyzed using the Una fold.rna platform (<http://unafold.rna.albany.edu/?q=mfold/RNA-Folding-Form>), where 2D structures were obtained. The structures are formed according to the rule of Gibbs free energy (ΔG).
